# The role of advanced glycation end-products in the development of coronary artery disease in patients with and without diabetes mellitus: a review

**DOI:** 10.1186/s10020-018-0060-3

**Published:** 2018-11-23

**Authors:** Sarah Louise Fishman, Halis Sonmez, Craig Basman, Varinder Singh, Leonid Poretsky

**Affiliations:** 1 Division of Endocrinology, Department of Medicine, Lenox Hill Hospital, Northwell Health, 110 East 59th St #8B, New York, NY 10022 USA; 2 Center for Diabetes and Endocrinology, 111 Salem Tpke, Norwich, CT 06360 USA; 3 Department of Cardiology, Lenox Hill Hospital, Northwell Health, 100 East 77th St, New York, NY 10065 USA

**Keywords:** Advanced glycation end-products (AGEs), Receptor for advanced glycation end-products (RAGE), Coronary artery disease (CAD), Diabetes mellitus (DM)

## Abstract

**Background:**

Traditional risk factors are insufficient to explain all cases of coronary artery disease (CAD) in patients with diabetes mellitus (DM). Advanced glycation end-products (AGEs) and their receptors may play important roles in the development and progression of CAD.

**Body:**

Hyperglycemia is the hallmark feature of DM. An increase in the incidence of both micro-and macrovascular complications of diabetes has been observed with increased duration of hyperglycemia. This association persists even after glycemic control has been achieved, suggesting an innate mechanism of “metabolic memory.” AGEs are glycated proteins that may serve as mediators of metabolic memory due to their increased production in the setting of hyperglycemia and generally slow turnover. Elevated AGE levels can lead to abnormal cross linking of extracellular and intracellular proteins disrupting their normal structure and function. Furthermore, activation of AGE receptors can induce complex signaling pathways leading to increased inflammation, oxidative stress, enhanced calcium deposition, and increased vascular smooth muscle apoptosis, contributing to the development of atherosclerosis. Through these mechanisms, AGEs may be important mediators of the development of CAD. However, clinical studies regarding the role of AGEs and their receptors in advancing CAD are limited, with contradictory results.

**Conclusion:**

AGEs and their receptors may be useful biomarkers for the presence and severity of CAD. Further studies are needed to evaluate the utility of circulating and tissue AGE levels in identifying asymptomatic patients at risk for CAD or to identify patients who may benefit from invasive intervention.

## Background

It has long been appreciated that age, gender, hyperlipidemia, hypertension, and smoking status contribute to the risk of developing coronary artery disease (CAD) (Goff Jr et al., 2013; McClelland et al., 2015). Concurrent diabetes mellitus (DM) is known to confer additional risk. Multiple studies have demonstrated that glucose intolerance, insulin resistance, and hyperglycemia are associated with coronary artery disease pathogenesis (Turner et al., 1998; de Vegt et al., 1999; DeFronzo & Ferrannini, 1991). Supporting the role of DM in the progression of CAD, prospective studies which included patients with DM treated intensively reported a significantly lower incidence of CAD long-term compared to those assigned to standard therapy. Interestingly, this reduction in the incidence and progression of CAD remained even after intensive treatment was stopped (Lancet, 1998; Nathan, 1993; Duckworth et al., 2009; Yamagishi et al., 2017).

Many studies have demonstrated that the beneficial effects of intensive glycemic control endure even after reversion to more relaxed blood glucose goals. The landmark studies “Diabetes Control and Complications Trial/Epidemiology of Diabetes Interventions and Complications” and “United Kingdom Prospective Diabetes Study” demonstrated reductions in diabetic micro- and macrovascular complications in patients with both type 1 and type 2 DM correlating with duration of intensive glycemic control (Diabetes, C., I. Complications Trial /epidemiology of diabetes, and G. complications study research, 2016; Diabetes et al., 2015; Martin et al., 2014; Holman et al., 2008a; Holman et al., 2008b). By contrast, in patients with long standing poorly controlled type 2 DM, intensive treatment did not reduce the risk of major cardiovascular events (Duckworth et al., 2009; Group, A.C, 2008; Action to Control Cardiovascular Risk in Diabetes Study, G, 2008; Saremi et al., 2010). Taken together, these studies suggest long term effects of glycemic control on the development and progression of diabetic complications. This idea of “metabolic memory” has been supported by animal studies demonstrating continued progression of diabetic retinopathy despite correction of hyperglycemia (Engerman & Kern, 1987). In rats with induced diabetes, animals with poor glycemic control after 6 months had sustained increases in markers of oxidative stress as compared to diabetic rats with good glycemic control initiated shortly after induction as well as non-diabetic control rats (Kowluru et al., 2004).

The mechanisms underlying metabolic memory remain incompletely understood. Many mediators of metabolic memory have been proposed, including advanced glycation end-products (AGEs), a class of molecules formed by non-enzymatic glycation of proteins, lipids, and nucleic acids. The formation of AGEs is enhanced in the presence of chronic hyperglycemia due to increased glucose availability. It has been hypothesized that early hyperglycemia leads to a proportional increase in AGE formation and oxidative stress. Over time, mitochondrial respiratory chain proteins become increasingly glycated and mitochondrial DNA damage occurs leading to a self-perpetuating cycle of AGE formation and oxidative stress independently of hyperglycemia (Testa et al., 2017).

AGEs have been linked to the aging process, the promotion of tumor metastasis, and the development of Alzheimer’s disease in addition to their role in the development of diabetic complications (Singh et al., 2014). These molecules may also play a role in the development of CAD, both independently and synergistically with DM (Piarulli et al., 2013; Kanauchi et al., 2001; Schalkwijk et al., 2004; Kralev et al., 2009). In this article, we review the nature of AGEs, their receptors, and the mechanisms by which they may contribute to the pathogenesis of CAD.

## Main text

### What are AGE’s?

Advanced glycation end-products (AGEs) are a heterogeneous class of endogenously produced or exogenously derived glycated proteins and lipoproteins. Endogenous AGE production occurs through the complex Maillard reaction in which reducing sugars undergo a series of non-enzymatic reactions leading to the development of reactive carbonyl compounds and the subsequent glycooxidation of proteins, lipids, and nucleic acids. Metabolism of glucose during glycolysis leads to production of methylglyoxal, a carbonyl intermediate in the production of certain AGEs. Under conditions of oxidative stress, reducing sugars, amino acids, and lipids undergo autooxidation to generate additional reactive carbonyl compounds and increase production of AGEs leading to tissue accumulation (Chappey et al., 1997; Bunn & Higgins, 1981; Singh et al., 2001; Ott et al., 2014).

The extent of AGE formation in vivo is proportional to the availability of substrate (i.e monosaccharides) as well as the rate of protein turnover. Long lived proteins with significant lysine and arginine content (for example collagen and elastin) are particularly susceptible to glycation. The normal physiological rate of AGE accumulation increases with advancing age, but is markedly increased in the presence of hyperglycemia, oxidative stress, and inflammation. AGE production and accumulation are stimulated by a variety of factors, including tobacco smoking, transitional metals, and reducing agents (Chappey et al., 1997; Singh et al., 2001; Nicholl & Bucala, 1998; Fleming et al., 2011; Cerami et al., 1997). Exogenous AGEs are found in high levels in the modern western diet, as a result of food processing methods including sterilization, microwaving, and grilling. This is consistent with the finding that circulating AGE levels are higher in the western world population (O'Brien & Morrissey, 1989; Vlassara & Uribarri, 2004).

AGEs can form on virtually all body proteins and accumulate at higher levels in long-lived tissues such as skin, crystalline lens, and glomerular basement membrane. There is considerable debate on the optimal approach to measuring AGEs in relation to clinical outcomes. Many studies report measurements of circulating AGE levels from peripheral blood samples. Urinary AGE levels measured using fluorescence have been shown to correlate with circulating levels as expected given renal excretion of AGEs in individuals with normal renal function. However, circulating proteins have a relatively short half-life in relation to structural proteins, and may therefore underestimate the accumulation of AGEs in tissue. Importantly, the development of diabetic complications occurs in long lived tissues, however measurement of AGEs from tissues often requires biopsy or invasive procedures to obtain sample material. More recently, methods have been developed to detect and measure AGEs with fluorescent properties in skin. This technique can be performed non-invasively by real-time measurement of autofluorescence on the skin of the inner side of an individual’s lower forearm (Meerwaldt et al., 2004; Fokkens & Smit, 2016).

Of the many known AGEs, *N*^*ϵ*^-(carboxymethyl)lysine (CML) and pentosidine are the best characterized. CML is a relatively inert molecule and is commonly used as an AGE marker in food analysis (Goldberg et al., 2004). Pentosidine is a ribose-derived glyco-oxidation product of arginine and lysine residues (Sell & Monnier, 1989); it is a well-accepted marker of cumulative protein damage in aging and a variety of disease states including DM (Sell et al., 1991). Both pentosidine and CML have fluorescent properties that allow for their detection in the circulation and in tissue.

### Pathological outcomes of AGE accumulation

As mentioned above, AGEs can accumulate in nearly every tissue including eye, kidney, liver, vasculature, reproductive tissues, muscle, bone, and brain. The increased concentration of AGEs in patients with DM may result from a cyclic process whereby glycated albumin disrupts normal glucose metabolism in muscle and adipocytes, leading to reduced insulin mediated glucose uptake and hyperglycemia (Unoki et al., 2007). Increased AGE levels have been associated with many microvascular diabetic complications (Genuth et al., 2015; Monnier et al., 2013); including retinopathy (Nagaraj et al., 2012; Genuth et al., 2005), nephropathy (Yamamoto et al., 2005; Makita et al., 1991), and neuropathy (Sugimoto et al., 2008; Araszkiewicz et al., 2011; Vouillarmet et al., 2013). Furthermore, the immunosuppressed state observed in patients with DM may be related to an excess of glycated immunoglobulins with disrupted functionality (Raghav et al., 2017). Increasing evidence points to a role for AGEs in the development of DM associated co-morbidities including non-alcoholic steatohepatitis (Hyogo & Yamagishi, 2008), osteoporosis (Wang et al., 2002) and polycystic ovarian syndrome (Merhi, 2014). Higher values of circulating CML levels and skin AGEs have been observed in patients with peripheral vascular disease and DM as compared to patients without DM (Raposeiras-Roubin et al., 2015; Bos et al., 2011; Arsov et al., 2014; de Vos et al., 2014). A causative role for AGEs in the pathogenesis of many of these outcomes is emerging.

Many studies have demonstrated an association between elevated AGE levels and cardiovascular disease in patients with DM. In a study of 339 patients with type 1 DM, the incidence of cardiovascular events correlated with baseline circulating AGE levels over a median follow-up period of 12 years. However, this association did not remain significant when patients with baseline nephropathy were excluded (Nin et al., 2011). Similarly, Koska et al. found a significant association between the incidence of cardiovascular events and baseline CML levels in a subgroup of participants from the ACCORD trial, but this finding was not significant when adjusted for a history of prior cardiovascular events (Koska et al., 2018). Two large studies have identified elevated circulating AGEs as an independent risk factor for cardiovascular mortality in women, but not in men (Kilhovd et al., 2007; Semba et al., 2009a). One prospective study of over 1000 adults over age 65 followed for a median time of 6 years demonstrated an association between higher circulating CML levels and cardiovascular mortality, which remained significant after adjustment for DM (Semba et al., 2009b). In patients with DM, elevated circulating pentosidine levels have also been associated with cardiovascular disease and were shown to correlate with increased arterial wall stiffness (Yoshida et al., 2005). Interestingly, in a study of patients with type 1 DM, levels of AGEs in the skin as measured by autofluorescence but not circulating CML levels were associated with increasing arterial wall stiffness (Llaurado et al., 2014). Skin autofluorescence has also been associated with macrovascular complications in patients with type 2 DM (Noordzij et al., 2012).

AGE accumulation has been associated with specific cardiac pathologies including congestive heart failure (Hartog et al., 2007), arrhythmias (Raposeiras-Roubin et al., 2012) and CAD (Kilhovd et al., 1999) in patients with DM. Elevated AGEs have been associated with both systolic and diastolic dysfunction in patients with DM (Hartog et al., 2007; Galderisi, 2006). AGE levels in patients with DM have been shown to correlate with the degree of systolic dysfunction (Steine et al., 2007) as well as indicators of diastolic dysfunction such as delayed relaxation time and end diastolic diameter (Kilhovd et al., 1999).

Many lines of evidence suggest that AGE levels may be useful as a biomarker for the presence and severity of CAD (Yeboah et al., 2004). In a study from Japan, circulating AGE levels were higher in patients with type 2 DM and obstructive CAD than in those with non-obstructive CAD (Kiuchi et al., 2001). This association was independent of other risk factors for CAD including smoking, hypertension, hyperlipidemia, and hyperuricemia. In a large study of 1320 patients with type 2 DM, Lu et al. demonstrated that elevated glycated albumin levels correlated with the severity of CAD as measured by quantitative coronary angiography (Lu et al., 2009). Circulating AGE levels have also been associated with in-stent restenosis risk in patient with DM (Choi et al., 2005; Lu et al., 2008; Shen et al., 2012). Skin autofluorescence is reportedly higher in patients with stable CAD as compared to healthy controls (Mulder et al., 2008).

More recently, AGEs have been implicated in contributing to cardiovascular mortality independently of DM (Semba et al., 2009a). Yozgatli et al., recently reported a correlation between increased tissue AGE levels as measured by skin autofluorescence and macrovascular events (including CAD, peripheral vascular disease, and cerebrovascular disease) independently of hemoglobin A1C measurement in a cohort of 563 subjects with type 2 DM (Yozgatli et al., 2018). In a small study of patients with confirmed normal glucose tolerance undergoing coronary angiography, circulating concentrations of AGEs were significantly higher in individuals with 3-vessel disease as compared to individuals with non-obstructive or single vessel disease (Kanauchi et al., 2001). This finding was supported by a subsequent study of 101 patients referred for coronary angiography in which increased circulating levels of pentosidine were associated with obstructive coronary disease, and were correlated with angiographic CAD severity, independently of DM status (Kerkeni et al., 2014). However, larger studies did not find an association between increased glycated albumin concentrations and CAD in patients without DM (Lu et al., 2009). Key studies supporting a role for AGEs in the development of CAD are summarized in Table 1.

**Table 1:** The advanced glycation end products (AGEs) and severity of coronary artery disease (CAD)

Reference	Type	(n)	Results
Kerkeni et al. (Kerkeni et al., 2014)	Randomized Control Trial	161	Serum pentosidine concentrations were significantly higher in patients with CAD in both patients with and without DM (*p* = 0.032 and 0.002, respectively). CML levels did not show a significant difference in patients with CAD between those with and without DM. The serum pentosidine concentrations were significantly higher in patients with CAD who had a Gensini score of > 20 compared to those with the score of “1–20” or “0” (*p* = 0.002 and *p* < 0.001, respectively). CML concentration was not associated with the severity of CAD (*p* = 0.853).
Lu L et al. (Lu et al., 2009)	Cross Sectional	1320	Elevated glycated albumin and reduced esRAGE levels correlated with the severity of CAD and progression of the plaque in patients with DM (*p* < 0.01). There were no significant differences in glycated albumin and esRAGE concentrations (in patients without DM) between patients with and without CAD.
Basta et al. (Basta et al., 2008)	Randomized Control Trial	81	AGE concentrations were significantly higher in patients with multi-vessel CAD compared to those with single vessel disease at both day 1 and day 180 after PCI (*p* =. 0.033 and 0.005, respectively), but not before PCI (p =. 0.60). There was a significant increase in sRAGE levels at 180 days *(*491 μg/ml [374–850]) compared to before and 1 day after PCI (406 μg/ml [266–575] and 393 μg/ml [222–554] respectively, *p* = 0.011). There was a correlation between CML levels and the extent of the stenting on day 1 and day 180 (*p* = 0.022 and *p* = 0.012, respectively).
Kiuchi et al. (Kiuchi et al., 2001)	Randomized Control Trial	83	AGE concentrations were significantly higher in patients with CAD who had DM compared to those without DM (2.8 vs. 5.5 mU/mL, respectively (*p* < 0.0125). However, AGE concentrations did not show a significant difference in patients without CAD between patients with and without DM. There was a significant association between AGE levels and severity of CAD in patients with DM (single vessel: 3.4 mU/mL, two vessels: 5.7 mU/mL, and three vessels: 7.2 mU/mL). There was no significant correlation between AGE levels and severity of CAD in patients with or without DM.
Kanauchi et al. (Kanauchi et al., 2001)	Observational	98	There were significantly higher AGE levels in patients with CAD and DM compared to control individuals (2.42 ± 0.65 vs. 1.96 ± 0.40 mU/mL, *p* < 0.01). The AGE concentrations significantly correlated with the severity of CAD (no CAD: 1.98 ± 0.29; 1 vessel: 2.09 ± 0.34; 2 vessels: 2.60 ± 0.73; and 3 vessels: 3.18 ± 0.58 mU/ml, *p* < 0.0001).

### Role of AGE receptors in pathological outcomes

AGEs can bind to a number of extracellular and intracellular proteins in a variety of cell types. Cell surface AGE receptors can be separated into two main types depending on the downstream effects of AGE binding an activation. Those involved in the endocytosis, breakdown, and removal of AGEs from the circulation; and those that activate a pro-inflammatory cellular response. AGER1, the prototype for the former class, has an additional role in inhibiting the production of reactive oxygen species and cellular defense mechanisms (Lu et al., 2004; Villegas-Rodriguez et al., 2016; Vlassara & Striker, 2011). AGER1 expression is upregulated on acute exposure to increased AGE concentrations, but is suppressed with chronic exposure to oxidative stress and high extracellular AGE levels, consistent with the finding of reduced AGER1 levels in patients with diabetes and chronic inflammatory disease (Vlassara & Uribarri, 2014). Additional cell surface receptors involved in reducing AGE concentrations include macrophage scavenger receptor I and II, oligosacharyltransferase-48, 80-KH phosphoprotein, CD36, galectin-3, and LOX-120, though these molecules have significantly weaker affinity for AGEs compared to AGER1.

**Fig. 1:**
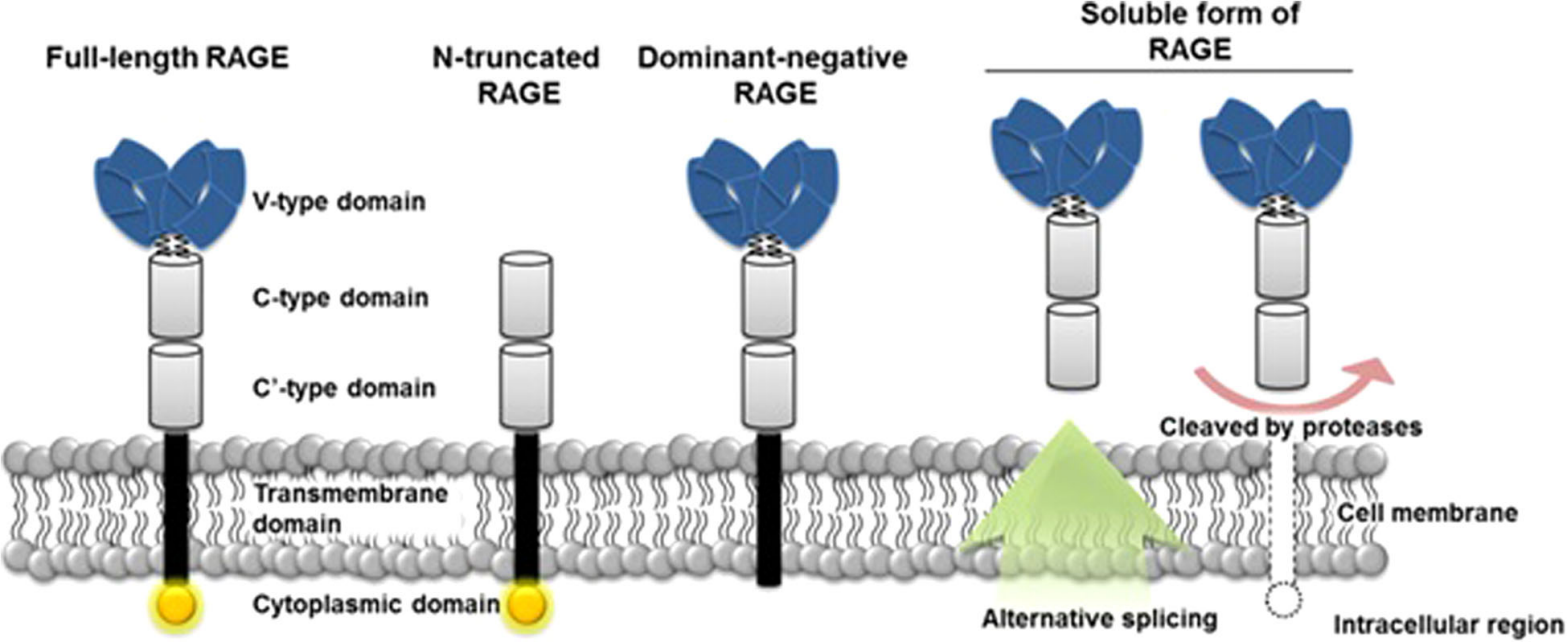
Major RAGE isoforms. The full length receptor includes one cytoplasmic domain involved in signal transduction, and three extracellular domains comprised of two c-type domains and one v-type domain. The N-truncated isoform lacks AGE binding properties and is not activated by ligand binding. Dominant negative RAGE serves as a cell-bound decoy receptor. It lacks a cytosolic domain and is not involved in signal transduction. Soluble RAGE consists of the complete extracellular domain, produced either via alternative splicing and directly secreted from the cell, or as a by-product of cleavage of full length RAGE by extracellular proteases. Copied with permission from Lee EJ, Park JH, Genomics and Informatics 2013

By contrast, receptor for AGE (RAGE), initiates complex signaling pathways when activated by AGE binding. RAGE belongs to the immunoglobulin superfamily of molecules and is comprised of a multi-ligand binding extracellular domain, a membrane spanning domain, and an intracellular carboxyl-terminal domain (Neeper et al., 1992). The extracellular domain is composed of three smaller domains, one V-type domain with homology to immunoglobulin variable domains, and two C-type domains with homology to the immunoglobulin constant domains. While RAGE is the product of a single gene, multiple alternative splice forms of RAGE exist leading to isoforms with partial functionality (Hudson et al., 2008) (Fig. 1). Three isoforms merit specific mention: N-truncated RAGE lacks an extracellular V-type domain, preventing binding of AGEs to the receptor; dominant negative RAGE lacks an intracellular domain, but remains anchored to the cell surface, serving as a decoy for AGE binding; and endogenous secreted RAGE (esRAGE), which lacks both a membrane spanning and an intracellular domain. Additionally, extracellular metalloproteinases can cleave the cytosolic portion of cell surface RAGE on endothelial cells leading to additional circulating receptor (Galichet et al., 2008). Along with esRAGE, these isoforms are collectively referred to as sRAGE. Because of their truncated structures, sRAGE molecules also serve as decoys for circulating AGEs and other ligands (Gkogkolou & Bohm, 2012).

RAGE is widely expressed, albeit at low levels, in a variety of cell types including macrophages, mesangial and mononuclear cells, smooth muscle cells, endothelial cells, certain neurons, hepatocytes, and podocytes (Mukherjee et al., 2005) with expression increasing in response to cellular stress (Goldin et al., 2006; Daffu et al., 2013). In addition, sRAGE is detectable in bodily fluids such as breast milk, saliva, tears, and nasal secretions (Schmidt et al., 1994).

It is hypothesized that reduced sRAGE levels and increased cell surface RAGE levels may also contribute to pathological outcomes. Lower concentrations of sRAGE have been reported in patients with DM compared to those without DM, and have been inversely correlated with HbA1C (Devangelio et al., 2007). Like AGEs, RAGE has been implicated in the development of diabetic micro- and macrovascular complications. Polymorphisms in the RAGE gene have been identified that are associated with increased risk of diabetic nephropathy (Prevost et al., 2005), and patients with type 2 DM with lower plasma sRAGE levels are more likely to have nephropathy and retinopathy (Grossin et al., 2008). Immunohistochemical studies of peripheral nerves in patients with diabetes demonstrated increased RAGE staining in patients with neuropathic symptoms (Juranek et al., 2013).

RAGE levels have also been associated with arterial stenosis and atherosclerosis both in patients with and without DM. Reduced esRAGE levels have been reported in patients with increased carotid artery intima media thickness (Katakami et al., 2005; Koyama et al., 2005), and sRAGE levels have been inversely correlated with the degree of atherosclerosis present in the carotid and femoral arteries (Falcone et al., 2005). Immunohistochemical analysis of carotid artery plaques recovered during endarterectomy demonstrated high positivity for cell surface RAGE (Cipollone et al., 2003).

The utility of sRAGE as a biomarker for cardiovascular disease has been studied with conflicting results. In multiple studies, sRAGE levels have been directly correlated with the presence and severity of CAD in patients both with and without DM (Kiuchi et al., 2001; Ha et al., 2013; Cai et al., 2011; Park et al., 2011; Jensen et al., 2015). However a large study of 1201 patients followed for 18 years reported that lower sRAGE levels increased the risk of DM, CAD, and all cause-mortality (Selvin et al., 2013). Many of these studies do not differentiate between esRAGE and soluble RAGE products resulting from cleavage of cell surface RAGE. Cell surface RAGE expression may be increased by binding and activation by AGEs. As such, high levels of sRAGE may reflect an increase in full length RAGE production, as opposed to an increase in esRAGE expression. Furthermore, some forms of sRAGE resulting from cleavage of full length RAGE may not be able to bind AGEs and serve as competitive inhibitors of cell surface RAGE. In addition, accepted normal values for sRAGE levels have not been established, as there is significant variability between populations and age groups (Wautier et al., 2017).

In a cohort of 154 patients, Wagner et al. reported that low plasma levels of esRAGE were associated with increased cardiovascular mortality in patients, suggesting that esRAGE is a more predictive biological marker than the cleaved isoforms of RAGE alone (Wagner et al., 2006). Neeper et al. (Neeper et al., 1992) demonstrated that increased levels of sRAGE along with reduced levels of esRAGE are associated with the development and progression of heart failure in patients with DM, and hypothesize that increased sRAGE levels result from increased metalloproteinase activity in patients with heart failure. Similarly, Yang et al. analyzed a cohort of 576 patients with type 2 DM and stable CAD undergoing PCI. They found that lower esRAGE levels were associated with a significantly higher rate of major cardiovascular events (Yang et al., 2015). Additional studies have also demonstrated an inverse correlation between esRAGE levels with severity of CAD and disease progression in patients with DM (Lu et al., 2009; Lu et al., 2008; Shen et al., 2012; Peng et al., 2009). Further studies are needed to evaluate esRAGE, as opposed to all soluble RAGE products, as a marker for coronary disease activity.

### Possible molecular mechanisms of AGE/RAGE mediated pathogenesis in cardiovascular disease

AGEs exert their pathogenic effects via three main molecular mechanisms: Modification of extracellular proteins, modification of intracellular proteins, and activation of signaling cascades via binding to cell surface RAGE. All three of these mechanisms may contribute to the development and progression of cardiovascular disease.

#### Extracellular protein modification

Modification of extracellular proteins by AGEs can alter the structure, function, and properties of normal tissue, as well as provoke an inflammatory response. Collagen, elastin, and laminin are key structural proteins of basement membrane and connective tissue. Given their long half-life and amino acid composition, these molecules are high susceptible to modification by AGEs. Glycated collagen molecules are resistant to proteolytic digestion (Bailey, 2001; Zieman & Kass, 2004), and form cross links with other extracellular proteins. This leads to decreased flexibility of vessel walls and vascular stiffness (Aronson, 2003). Glycation of structural extracellular proteins in the myocardial matrix will similarly increase myocardial stiffness, contributing to impaired relaxation and diastolic dysfunction (Candido et al., 2003). In addition to glycated collagen, glycation of elastin and laminin in basement membrane have also been shown to impair endothelial cell adhesion and migration by disrupting cell attachment sites (Haitoglou et al., 1992). These alterations in cell-matrix interactions are associated with a reduction in stress-induced nitric oxide production by endothelial cells and impaired vasodilation.

Glycation of additional circulating factors contributes to thrombogenesis, hypercoagulability, and decreased fibrinolysis. Glycated fibrinogen is significantly more resistant to degradation (Takenaka et al., 2006; Murakami et al., 1990) and modification of annexin II and heparin cofactor II renders these proteins dysfunctional, impairing fibrinolytic systems (Takenaka et al., 2006; Gugliucci & Ghitescu, 2002; Ceriello et al., 1990). Glycated low density lipoproteins (LDL) (Zoltowska et al., 2004) and platelet glycoproteins (Winocour et al., 1992) can enhance platelet sensitivity to aggregating agents, enhancing thrombogenesis. In addition, glycated LDL has been shown to decrease tissue plasminogen activator production in endothelial cells (Zhang et al., 1998). Extracellular protein glycation can also directly promote atherosclerosis. Glycation of LDL molecules alters their structure, inhibiting their uptake by LDL receptors and clearance from the circulation, allowing enhanced uptake by monocytes and macrophages, promoting foam cell generation (Cai et al., 2004; Sobal et al., 2001).

#### Intracellular protein modification

Intracellular accumulation of AGEs occurs in the endoplasmic reticulum, leading to stress which can impair normal protein folding processes. Cellular mechanisms exist to identify improperly folded proteins and to activate the unfolded protein response leading to cell apoptosis (Adamopoulos et al., 2014). Intracellular AGEs can bind to mitochondrial proteins involved in electron transport, decreasing ATP synthesis and increasing superoxide and reactive oxygen species production. Furthermore, glutathoine peroxidase and glutathione reductase, enzymes of the antioxidant system, can be modified by AGEs leading to decreased enzymatic activity. In cardiomyocytes, crosslinking of intracellular glycated ryanodine receptors and SERCA2a alters calcium homeostasis, reducing the contractility of the tissue and contributing to the development of systolic heart failure (Bidasee et al., 2003; Bidasee et al., 2004).

#### AGE mediated signaling cascades

Binding of AGE to full length RAGE activates many signaling cascades, ultimately resulting in the generation of pro-inflammatory mediators and reactive oxygen species, and stimulation of proliferative, fibrotic, and thrombotic pathways leading to vascular inflammation (Prasad et al., 2012; Brownlee et al., 1988). RAGE contains a multi-ligand binding extracellular domain with affinity for multiple AGEs as well as S100, amyloid, and fibrillar protein aggregates, linking RAGE mediated signaling to a number of pathogenic processes including neurodegeneration, amyloidosis, and tumor growth. By contrast, AGE binding to cell surface AGER1 inhibits these processes by suppressing and disrupting RAGE signaling. In this way, decreased levels of AGER1, may contribute to pathogenic outcomes.

The RAGE gene promoter region contains an NFκβ binding domain, suggesting that expression of RAGE is upregulated as part of the inflammatory response. This creates a positive feedback loop, as activation of RAGE by AGEs leads to a series of phosphorylation reactions, including MAPK activation, and results in translocation of NFκβ to the cell nucleus and enhanced expression of additional pro-inflammatory cytokines and proteins including ras, IL-6, TNFα, TGF-β, and vascular adhesion molecules (VCAM-1, ICAM-1, endothelin-1) (Ramasamy et al., 2009; Schmidt et al., 2001; Ramasamy et al., 2005a; Ramasamy et al., 2005b). RAGE activation also enhances activity of the Jak/Stat signaling pathway and upregulation of interferon responsive genes (Ott et al., 2014).

AGE/RAGE interactions lead to activation of NADPH and nitric oxide synthase (via NFκβ mediated upregulation) perpetuating a cycle of reactive oxygen species production, continued enzyme activation, and stimulation of NFκβ (Ott et al., 2014). Furthermore, AGEs can directly inactivate nitric oxide (Hogan et al., 1992), which at normal low intracellular concentrations functions as an anti-oxidant, anti-proliferative, and anti-thrombotic agent and is an important mediator of vasodilation (Stitt et al., 2002). Reduced concentrations of nitric oxide allow for an increase in the formation of reactive oxygen and reactive nitrogen species, stimulating the cellular oxidative stress reaction (Schmidt et al., 1994; Schreck et al., 1991). The oxidative stress generated from the AGE/RAGE interaction can also lead to vascular smooth muscle apoptosis which contributes to calcifications in the vessel walls (Prasad et al., 2012). This pathway may explain the transition of bovine smooth muscle cells to osteoblast like cells when cells are grown in hyperglycemic versus euglycemic conditions (Chen et al., 2006).

**Fig. 2:**
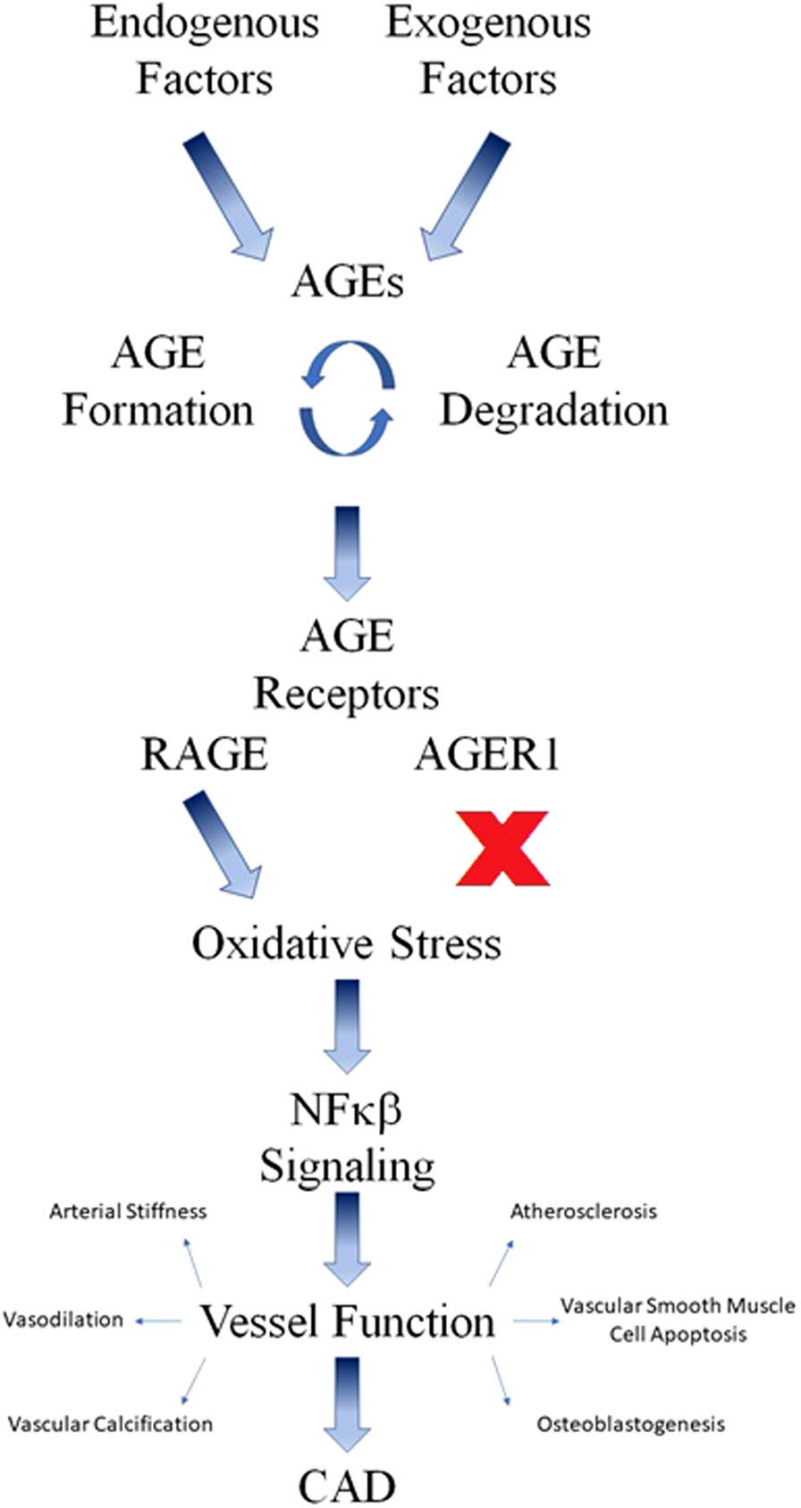
AGE signaling cascade implicated in the development of CAD. Both endogenous and exogenously derived AGEs are involved in a cycle of formation and degradation. AGEs are recognized by two different classes of receptors which either activate or suppress the generation of reactive oxidative species. Oxidative stress leads to activation of NFκβ with multiple effects on vessel function which contribute to the development of CAD

Figure 2 summarizes the signaling cascade involving AGEs and their possible relationship to CAD.

### AGEs as a therapeutic target

Multiple in-vitro and animal studies have demonstrated a beneficial effect of reducing AGE levels and AGE/RAGE pathway activation in preventing and halting the development of DM complications including cardiovascular disease. Multiple compounds have been shown to reduce the accumulation of AGEs either by blocking formation or by increasing removal. Aminoguanidine and pyridoxamine have anti-oxidant properties that inhibit AGE formation. While aminoguanidine treatment led to improvements in vascular and myocardial function in rats (Chang et al., 2006) and reduced atherosclerotic plaque area in mice (Forbes et al., 2004), clinical trials of aminoguanidine in humans failed to show significant improvements in diabetes complications and were hampered by safety concerns (Bolton et al., 2004; Freedman et al., 1999). In one study, 212 subjects with diabetes and nephropathy treated with pyridoxamine, a vitamin B6 analog, showed improved serum creatinine levels with minimal adverse effects (Williams et al., 2007). Benfotiamine, a lipophilic thiamine analog, reduces production of AGEs by shunting monosaccharide substrates to the pentose phosphate pathway via activation of the enzyme transketolase (Goh & Cooper, 2008; Huijberts et al., 2008). Clinical studies to investigate the efficacy of benfotiamine in reducing diabetic complications have yielded conflicting results (Sanchez-Ramirez et al., 2006; Stracke et al., 2001; Alkhalaf et al., 2010; Alkhalaf et al., 2012; Fraser et al., 2012) Alagebrium disrupts existing AGEs by cleaving carbon-carbon bonds in carbonyl groups. Small clinical trials suggested a therapeutic potential for this drug in improving cardiac dysfunction and diabetic renal disease (Coughlan et al., 2007). However subsequent larger trials were not able to replicate these findings, and failed to show an improvement in AGE accumulation (Willemsen et al., 2010; Nenna et al., 2015).

Blockade of cell surface RAGE results in inhibition of the pro-inflammatory effects of AGEs (Hori et al., 1996; Schmidt et al., 1999). Rats and mice treated with sRAGE infusions or RAGE inhibitors have shown reduced rates of atherosclerosis (Ha et al., 2013; Wautier et al., 1996; Bucciarelli et al., 2002; Soro-Paavonen et al., 2008). Gene knockout studies in mice have also shown reductions in atherosclerosis, oxidative stress, and inflammation in mice lacking the RAGE gene (Yan et al., 1994). Furthermore, cardiomyocytes obtained from RAGE knockout mice are protected from cellular damage (Shang et al., 2010). Some of the currently available therapies developed to treat DM and CAD have been shown to impact the AGE/RAGE axis. Tam et al. reported an increase in esRAGE levels in patients with DM treated with atorvastatin, with a correlating decrease in LDL levels (Tam et al., 2010). In a rat model, atorvastatin treatment for 24 weeks increased the serum and renal sRAGE levels and decreased renal RAGE expression in rats with DM, resulting in reduced accumulation of AGEs (Lu et al., 2011). Rosiglitazone, an oral insulin sensitizing agent, has been shown to reduce esRAGE levels with 6 months of treatment (Tan et al., 2007). In addition, both metformin and pioglitazone can block AGE formation in vitro *(Rahbar et al.,* 2000*)*. ACE inhibitors and angiotensin receptor blockers have also been hypothesized to have AGE formation blocking activity (Miyata et al., 2002). Some evidence suggests GLP-1 agonists reduce RAGE expression (Yamagishi et al., 2015; Yamagishi & Matsui, 2011). DPP4 inhibitors may protect against diabetic nephropathy by suppressing activation of the AGE-RAGE axis (Nakashima et al., 2014).

Finally, reduced consumption of AGEs from the diet can significantly reduce systemic inflammation in humans despite endogenous AGE production being the major source of circulating AGEs (Vlassara et al., 2002). Sevelamer carbonate, a compound frequently used in patients with advanced kidney disease as a phosphate binder, is a non-absorbed oral agent that can bind AGEs and may reduce intestinal absorption. In one study of 117 patients with type 2 DM and chronic kidney disease, administration of sevelamer carbonate was shown to reduce circulating levels of CML and full length RAGE, and increase AGER1 levels independently of a reduction in hemoglobin A1C (Yubero-Serrano et al., 2015; Vlassara et al., 2012).

## Conclusions

Increasing evidence supports a role for the AGE/RAGE axis in the development, severity, and progression of CAD in patients with and without DM. Additional prospective multicenter randomized controlled studies are needed to further evaluate the possibility that circulating AGE or sRAGE levels can serve as a biomarker to diagnose CAD and to identify patients who may benefit from invasive intervention for diagnosis and treatment. The literature to date evaluating these questions has been limited by differences in techniques for AGE and RAGE quantification, and inconsistent measurements of specific molecules. There is increasing evidence supporting a role for AGEs and AGE/RAGE signaling in the development of CAD and other diabetic complications. Development of therapeutic agents aimed at reducing circulating AGE concentrations and blocking of RAGE activation may reduce the complications of DM and aid in the treatment of CAD.

## Abbreviations

AGEs: Advanced glycation end-products
CAD: Coronary artery disease
CML: Carboxymethyllysine
DM: Diabetes mellitus
LDL: Low density lipoporteins
RAGE: Receptor for advanced glycation end-products
sRAGE: Soluble receptor for advanced glycation end-products
